# The Effects of Ginsenoside Compound K Against Epilepsy by Enhancing the γ-Aminobutyric Acid Signaling Pathway

**DOI:** 10.3389/fphar.2018.01020

**Published:** 2018-09-11

**Authors:** Xiangchang Zeng, Kai Hu, Lulu Chen, Luping Zhou, Wei Luo, Chaopeng Li, Wenjing Zong, Siyu Chen, Qing Gao, Guirong Zeng, Dejian Jiang, Xiaohui Li, Honghao Zhou, Dong-sheng Ouyang

**Affiliations:** ^1^Department of Clinical Pharmacology, Xiangya Hospital, Central South University, Changsha, China; ^2^Institute of Clinical Pharmacology, Central South University, Changsha, China; ^3^Department of Neurology, Xiangya Hospital, Central South University, Changsha, China; ^4^Hunan Key Laboratory of Pharmacodynamics and Safety Evaluation of New Drugs & Hunan Provincial Research Center for Safety Evaluation of Drugs, Changsha, China; ^5^Department of Pharmacology, School of Pharmaceutical Sciences, Central South University, Changsha, China; ^6^Hunan Key Laboratory for Bioanalysis of Complex Matrix Samples, Changsha Duxact Biotech Co., Ltd., Changsha, China

**Keywords:** ginsenoside compound K, epilepsy, neurotransmitters, GABA_A_Rα1, KCC2, NKCC1

## Abstract

The imbalance between the GABA-mediated inhibition and the glutamate-mediated excitation is the primary pathological mechanism of epilepsy. GABAergic and glutamatergic neurotransmission have become the most important targets for controlling epilepsy. Ginsenoside compound K (GCK) is a main metabolic production of the ginsenoside Rb1, Rb2, and Rc in the intestinal microbiota. Previous studies show that GCK promoted the release of GABA from the hippocampal neurons and enhanced the activity of GABA_A_ receptors. GCK is shown to reduce the expression of NMDAR and to attenuate the function of the NMDA receptors in the brain. The anti-seizure effects of GCK have not been reported so far. Therefore, this study aimed to investigate the effects of GCK on epilepsy and its potential mechanism. The rat model of seizure or status epilepticus (SE) was established with either Pentylenetetrazole or Lithium chloride-pilocarpine. The Racine’s scale was used to evaluate seizure activity. The levels of the amino acid neurotransmitters were detected in the pilocarpine-induced epileptic rats. The expression levels of GABA_A_Rα1, NMDAR1, KCC2, and NKCC1 protein in the hippocampus were determined via western blot or immunohistochemistry after SE. We found that GCK had deceased seizure intensity and prolonged the latency of seizures. GCK increased the contents of GABA, while the contents of glutamate remained unchanged. GCK enhanced the expression of GABA_A_Rα1 in the brain and exhibited a tendency to decrease the expression of NMDAR1 protein in the hippocampus. The expression of KCC2 protein was elevated by the treatment of GCK after SE, while the expression of NKCC1 protein was reversely down-regulated. These findings suggested that GCK exerted anti-epileptic effects by promoting the hippocampal GABA release and enhancing the GABA_A_R-mediated inhibitory synaptic transmission.

## Introduction

Epilepsy is a common chronic neurological disease, characterized by the presence of spontaneous unprovoked recurrent seizures (Hauser et al., 2017). It affects over 70 million people around the world and approximately 2.4 million persons are diagnosed with epilepsy each year (Yemadje et al., 2011; van Vliet et al., 2017). Epilepsy appears from a variety of complex causes, such as febrile seizures, head trauma, birth injuries, stroke, brain tumor, infections, and genetics (Singh and Trevick, 2016). Epilepsy comprises numerous seizure types and syndromes, where it easily coexists with psychiatric and neurological comorbidities (Kanner, 2016). A number of patients diagnosed with new onset epilepsy obtain symptomatic remission with the use of anti-epileptic drugs (Brodie et al., 2012). However, 20–30% of patients are ineffective in the currently available anti-epileptic drugs (Sillanpaa and Schmidt, 2006). Nearly half of patients will suffer mild, moderate, or severe adverse reactions (Schmidt and Schachter, 2014). There is an urgent need to develop a novel high efficiency and low toxic anti-epileptic drug for the treatment of intractable epilepsy.

The imbalance between excitatory and inhibitory neurotransmission is known to be one of the most important causes of seizures (Margineanu and Klitgaard, 2009; Amtul and Aziz, 2017). Glutamate is the primary excitatory neurotransmitter in the brain. Glutamate acts on its postsynaptic receptors to mediate excitatory neurotransmission, which is involved in neural development and synaptic plasticity (Guerriero et al., 2015). However, large amounts of glutamate are released from presynaptic neurons under pro-epileptogenic stimuli, including status epilepticus (SE), stroke, and traumatic brain injury (Wang et al., 2012). Excessive glutamate in the synaptic cleft induces excitotoxicity by activating the NMDAR, which promotes calcium influx that leads to neuronal death (Wang and Qin, 2010). NMDAR activation also promotes limbic epileptogenesis by enhancing the synaptic excitation (McNamara et al., 2006). MK-801, a NMDA receptor antagonist, inhibits seizure activity in the amygdala kindling model of epilepsy (Sato et al., 1988). Suppressing the glutamate-NMDAR pathway could inhibit the occurrence of epilepsy.

γ-aminobutyric acid (GABA) is the main inhibitory neurotransmitter in the mammalian central nervous system. GABA acts on the GABA-A receptor after being released from the presynaptic vesicles, which promotes the opening of the Cl^-^ channels and causes hyperpolarization of the postsynaptic cell (Guerriero et al., 2015). Emerging evidence shows that GABA_A_Rα1 was reduced in pilocarpine-induced epileptic rats (Raol et al., 2006b). The enhanced GABA_A_Rα1 expression could increase the seizure threshold and inhibit the development of recurrent spontaneous seizures after SE, suggesting that GABA_A_Rα1 plays a major role in inhibitory function (Raol et al., 2006a). The GABA_A_R agonist diazepam is a classical anti-seizure drug that is used for treating SE (Levi et al., 2015), suggesting that the GABA_A_R-mediated inhibitory neurotransmission is a therapeutic target for epilepsy. The GABA_A_ receptors subunit composition affects the function of GABAergic inhibition, the intracellular Cl^-^ concentration is a critical determinant of postsynaptic inhibition. The expression patterns, membrane trafficking, and protein degradation of cation-chloride cotransporters control the Cl^-^ levels in neurons (Loscher et al., 2013). The NKCC1 promotes the Cl^-^ influx while the KCC2 extrudes Cl^-^. The dysfunction of NKCC1 and KCC2 enhances neuronal excitability and promotes susceptibility to seizures. Restoring Cl^-^ homeostasis could reduce the seizure severity by the NKCC1 inhibitor bumetanide or optogenetic removal of Cl^-^ (Moore et al., 2017), suggesting that NKCC1 and KCC2 are the key regulators of GABAergic inhibition which are potential targets for the treatment of epilepsy.

Ginsenoside compound K (GCK), also known as M1 or IH901, is a main metabolic product from Ginsenoside Rb1, Rb2, and Rc in intestinal bacteria following oral administration of ginseng (Lee et al., 2009). GCK has attracted a wide attention because of its good bioavailability (Paek et al., 2006). Several studies demonstrated that GCK exhibited multiple pharmacological activities including anti-tumor, anti-diabetic, anti-inflammation and hepatoprotective effects (Yang et al., 2015). GCK has not been reported as a new drug that is approved to enter the market. GCK has become a candidate drug for rheumatoid arthritis therapy due to its strong anti-inflammatory effect. At present, the GCK tablet was produced by Hisun Pharmaceutical Co., Ltd., which is being tested as an anti-rheumatoid arthritis drug in China. Our previous research has shown that GCK is safe and well-tolerated for healthy Chinese volunteers, where it exhibits a good pharmacokinetic profiles at a moderate dose (Chen et al., 2018). Its pharmacokinetic properties are affected by food and gender in humans (Chen et al., 2017). These findings indicated that GCK has a promising druggability that provides guidance for the development of CK. The research regarding the role of GCK in the nervous system has gradually increased. A previous study using quantitative autoradiography found that GCK decreased the binding of [^3^H]MK-801 with the NMDA receptor in the frontal cortex and hippocampus and enhanced the binding of [^3^H]muscimol and the GABA_A_ receptor in the frontal cortex and cerebellum. This suggested that GCK may suppress the activity of NMDA receptor and increase the effect of GABA receptor agonist in brain which plays an important role in neurological disorders (Jang et al., 2004). GCK could also enhance the spontaneous GABA release into the CA3 pyramidal neurons to induce inhibitory transmission (Bae et al., 2010). A study reported that GCK inhibits glutamate-induced cytotoxicity in hippocampal HT22 cells by regulating the Nrf2-mediated induction of antioxidant enzymes (Seo et al., 2016). GCK could also decrease the morphine-induced NMDAR1 activation in cultured cortical neurons (Yayeh et al., 2016). These findings suggest that GCK might reduce neuronal hyperexcitability by correcting the neuronal excitation-inhibition imbalance.

Since GCK may regulate the GABA receptor activity and the NMDA receptor expression, we hypothesized that GCK could have an anti-epileptic effect. Therefore, the purpose of this study is to investigate the effects of GCK in epilepsy and its potential mechanisms. Two classical epileptic animal models were established to evaluate the anti-seizure activity of GCK. We found that GCK exhibited a good anti-epileptic effect via enhancing the GABA-mediated inhibition in the hippocampus, which may possess a promising future for development of a novel anti-epileptic drug.

## Materials and Methods

### Reagents

Ginsenoside compound K was provided from Zhejiang Hisun Pharmaceutical Company Limited (China). Sodium valproate was obtained from Hunan Xiangzhong Pharmaceutical Company Limited (China). Pentylenetetrazole, Lithium chloride, Pilocarpine and paraformaldehyde were purchased from Sigma (United States).

### Animal Allocation and Drug Administration

Adult male Sprague–Dawley rats (6–8 weeks old, 180–200 g body weight) were purchased from Hunan SJA Laboratory Animal Co. Ltd. (China). Rats were housed in clear cages, 3 per cage. The experimental room was maintained in 22–23°C with humidity of 10–55%, it was kept on 12 h light or dark cycles. All rats access to food and water throughout the experiment. All experimental protocols were approved by the Ethics Committee of Drug safety evaluation research center of Hunan province and performed in accordance with the National Institute of Health Guide for the Care and Use of Laboratory Animals. All efforts were made to minimize the animal’s suffering.

After a week of acclimatization, 60 rats were randomly grouped into control, model, the positive control (Sodium valproate, 400 mg/kg), the GCK low (80 mg/kg), the middle (160 mg/kg), or the high dose (320 mg/kg) with 10 rats per group. The GCK suspension was prepared with the solution of sodium carboxymethyl cellulose (0.5% CMC-Na solution) prior to administration. Rats in the experimental group were given the corresponding drug via gavage twice a day in dosing intervals every 12 h for 5 days while the other rats were treated with the same dose of physicological saline. Rats were treated with pentylenetetrazole or lithium chloride-pilocarpine to initiate an epilepsy model 1 h after the last administration of GCK or Sodium valproate.

### Pentylenetetrazole-Induced Seizures

Pentylenetetrazole (PTZ) is a GABA_A_ receptor antagonist that is commonly used to establish tonic-clonic seizures and screen anti-seizure drugs. We choose this animal model to examine the anti-seizure activity of GCK. The seizure rat model was induced by injecting 60 mg/kg of PTZ. The seizure activity was immediately evaluated within 30 min after PTZ administration according to the modified Racine scale (Sadek et al., 2016): stage 0, inactive; stage 1, ear and facial twitching; stage 2, convulsive wave through the body; stage 3, myoclonic jerks and rearing; stage 4, turn over into side position and stage 5, turn over into back position, generalized clonic-tonic seizures. The time from injecting PTZ to the first appearance of convulsive wave through the body was measured for each animal and was referred to as the seizure latency. The total duration of the behavioral seizure activity was measured for each animal.

### Lithium Chloride-Pilocarpine-Induced Status Epilepticus (SE)

Pilocarpine is an M-receptor agonist, used as a convulsant to induce SE or TLE in animal models. Lithium chloride potentiates the epileptogenic action of pilocarpine and reduces mortality rates. Here we built a rat model of SE with Lithium chloride-pilocarpine. Rats were treated with intraperitoneal injection of pilocarpine (30 mg/kg, i.p.) 18–20 h after the lithium chloride (127 mg/kg, i.p.) injection. Scopolamine methyl bromide (1 mg/kg, i.p.) was administered to reduce peripheral adverse reactions. Rats were continuously observed 2 h following the injection of pilocarpine. The evoked seizures were assessed according to Racine scale (Inoue et al., 2009): 0, no abnormality; 1, mouth and facial movements; 2, head nodding; 3, forelimb clonus; 4, rearing and bilateral forelimb clonus; 5, rearing, falling and jumping. The time from the pilocarpine injection to the first appearance of the forelimb clonus was measured for each animal and was referred to as the seizure latency. Rats were euthanized by being anesthetized with 3 ml/kg of chloral hydrate. The entire brain post-SE 24 h was collected for immunohistochemical analysis. The dissociated hippocampus post-SE 3 h was used to determine the neurotransmitters. The hippocampus post-SE 24 h was obtained for western-blot analysis.

### Quantitative Analysis for Glutamate and GABA

To determine glutamate and GABA in the hippocampus, the hippocampus tissue was homogenized in an ice-cold PBS buffer and centrifuged at 10,000 rpm for 10 min at 37°C. The supernatant was used in the following assays.

The concentration of glutamate in the hippocampus was tested via the ultraviolet colorimetry method according to the instructions in the Glutamic acid measurement kit (Nanjing Jiancheng Bioengineering Institute, China).

The content of GABA was measured with the ELISA Kit for Gamma-Aminobutyric Acid (Cloud-Clone Corp., United States) in accordance with the manufacturer’s instructions. The sample was added into the prepared Detection Reagent A and incubated for 1 h at 37°C. The unbound conjugate was washed off, each microplate well was added to the prepared Detection Reagent B, and subsequently incubated for 30 min at 37°C. The substrate solution and the stop solution were used for color development reaction and termination. The absorbance was measured at 450 nm with a microplate reader (Beckman Coulter, United States). Each experiment was repeated in triplicate.

### Western-Blot Analysis

The frozen hippocampus was homogenized in the RIPA lysis buffer prepared with Phenylmethylsulfonyl fluoride (PMSF). The mixture was centrifuged at 12,000 rpm for 15 min at 4°C and the supernatant was collected. The content of total protein was measured with a BCA protein assay kit (Beyotime Biotechnology, China). 50 μg of the protein sample was added and separated via SDS-polyacrylamide gel electrophoresis. The blots were blocked after being transferred onto the PVDF membrane. The membrane was incubated overnight at 4°C with the primary antibodies: the mouse anti-GABA_A_Rα1 monoclonal antibody (1:500, Abcam, United Kingdom), the rabbit anti-NMDAR1 polyclonal antibody (1:500, Sigma, United States), the goat anti-NKCC1 polyclonal antibody (1:200, Santa Cruz, United States), goat anti-KCC2 polyclonal antibody (1:200, Santa Cruz, United States), and the rabbit anti-β-actin polyclonal antibody (1:1000, CST, United States). The blots were then incubated in the secondary antibodies: the HRP-labeled goat anti-mouse IgG, the goat anti-rabbit IgG, or the mouse anti-goat IgG (Beyotime Biotechnology, China) for 1 h at room temperature. The bands were visualized with an ECL chemiluminescence substrate kit (Beyotime Biotechnology, China) and scanned. The OD value was analyzed with ImageJ 1.50i software (United States).

### Immunohistochemical Staining

Rats were anesthetized and perfused transcardially with an ice-cold phosphate buffer, followed by an ice-cold 4% paraformaldehyde solution. The entire brain was removed immediately and immersed in 4% paraformaldehyde solution for 24 h at 4°C. The coronal sections were obtained through the dorsal hippocampus and used for immunohistochemical analysis.

The sections dewaxed and hydrated, then incubated in 3% hydrogen peroxide solution for 30 min. Antigen retrieval was performed under boiling conditions. Sections were incubated in goat serum for 2 h to block the antigens. Sections were incubated overnight at 4°C with the primary antibodies: the mouse anti-GABA_A_Rα1 monoclonal antibody (1:100, Abcam, United Kingdom), the goat anti-NKCC1 polyclonal antibody (1:50, Santa Cruz, United States), and the goat anti-KCC2 polyclonal antibody (1:50, Santa Cruz, United States). The sections were then incubated in the secondary antibodies: the biotinylated-goat anti-mouse IgG, or the mouse anti-goat IgG (Beyotime Biotechnology, China) for 1h at room temperature. After being rinsed three times with PBS, a DAB kit was used to visualize the sites of antibody binding. The sections were observed under a microscope. The positive cells in the hippocampal CA1, CA3, DG, and H region were captured. Three high-power images were randomly selected for each animal. The immunoreactivity was evaluated with the staining intensity and the ratio of positive area to the total area.

### Statistical Analysis

All experimental data was expressed as Mean ± Standard Error of the Mean (SEM), SPSS19.0 software was used for statistical analysis. The seizure score was analyzed by the Mann–Whitney *U* test. Seizure latency, duration, Glutamate, GABA and OD values were subjected to one-way ANOVA and *post hoc* comparisons were performed with an LSD test. *P* < 0.05 was considered statistically significant.

## Results

### Effects of GCK on Behavioral Seizures Induced by Pentylenetetrazole

**FIGURE 1:**
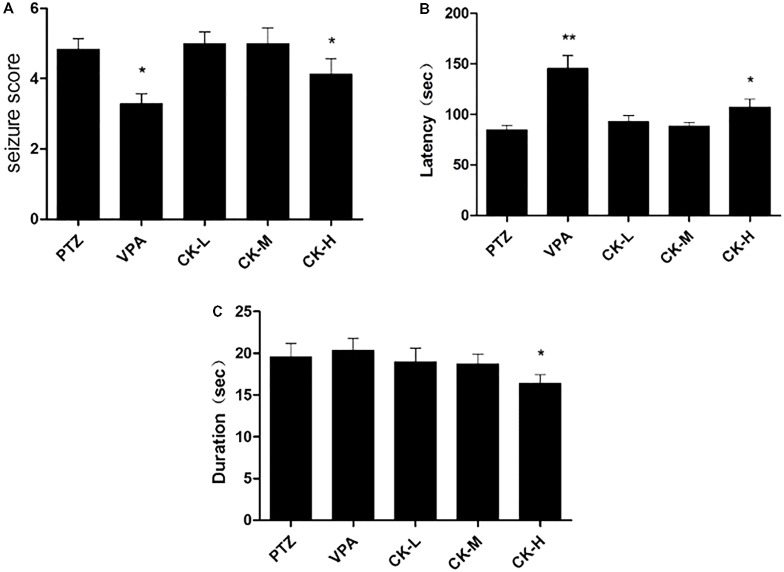
Effects of GCK on behavioral seizures induced by pentylenetetrazole (PTZ). **(A)** The seizure scores of the PTZ, VPA, and GCK-treated groups. **(B)** The latency to clonic seizures in the PTZ, VPA, and GCK-treated groups. **(C)** The duration of the PTZ, VPA, and GCK-treated groups. Values are mean ± SEM (*n* = 10). PTZ 60 mg/kg, VPA 400 mg/kg, GCK 80 mg/kg, 160 mg/kg, and 320 mg/kg. Values were compared with the PTZ, ^∗^*P* < 0.05, ^∗∗^*P* < 0.01.

To investigate the protective effect of GCK in an acute seizure animal model, 60 mg/kg of PTZ was administered to establish a seizure rat model that could replicate generalized tonic-clonic and myoclonic seizures. Rats in the model group showed an obvious epileptic behavioral feature after the PTZ injection. Rats pre-treated with VPA had a lower seizure score (**Figure 1A**, *P* < 0.05) and a longer latency than the model group (**Figure 1B**, *P* < 0.01). Unfortunately, GCK did not display a protective effect in the low-dose group and the middle-dose group. Interestingly, high doses of GCK not only reduced the seizure intensity (**Figure 1A**, *P* < 0.05) but also prolonged the latency for the onset of seizures (**Figure 1B**, *P* < 0.05). Moreover, high doses of GCK could shorten seizure duration (**Figure 1C**, *P* < 0.05). These findings suggest that high doses of GCK demonstrate anti-epileptic activity.

### Effects of GCK on Behavioral Seizures Induced by Lithium Chloride-Pilocarpine

**FIGURE 2:**
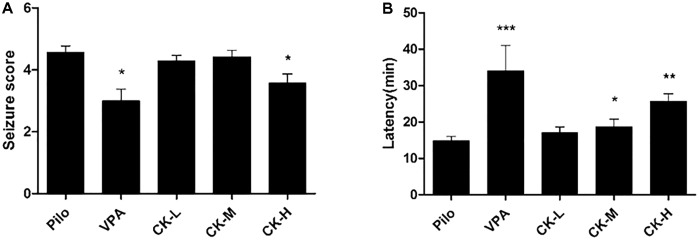
Effects of GCK on behavioral seizures induced by lithium chloride-pilocarpine. **(A)** The seizure scores of the Pilo, VPA, and GCK-treated groups. **(B)** The latency to onset SE in the Pilo, VPA, and GCK-treated groups. Values are mean ± SEM (*n* = 10). Pilo 30 mg/kg, VPA 400 mg/kg, GCK 80 mg/kg, 160 mg/kg, and 320 mg/kg. Compared with the Pilo, ^∗^*P* < 0.05, ^∗∗^*P* < 0.01, ^∗∗∗^*P* < 0.001.

To further identify GCK’s antiepileptic activity, the lithium chloride-pilocarpine-induced SE rat model was selected. This model resembled human SE and complex partial seizures. All rats in the model group exhibited high seizure scores (forelimb clonus, rearing, falling and jumping, even death). Seizure score was decreased in the VPA group (**Figure 2A**, *P* < 0.05) while the latency to the first seizure was extended in the VPA group (**Figure 2B**, *P* < 0.001). High doses of GCK significantly reduced the seizure score (**Figure 2A**, *P* < 0.05). Intriguingly, both middle and high doses of GCK significantly lengthened the latency to the initial seizure (**Figure 2B**, *P* < 0.05, 0.01). These results further confirmed that GCK has an anti-epileptic effect.

### Effects of GCK on the Contents of Amino Acid Neurotransmitter in Pilocarpine-Induced Epileptic Rats

**FIGURE 3:**
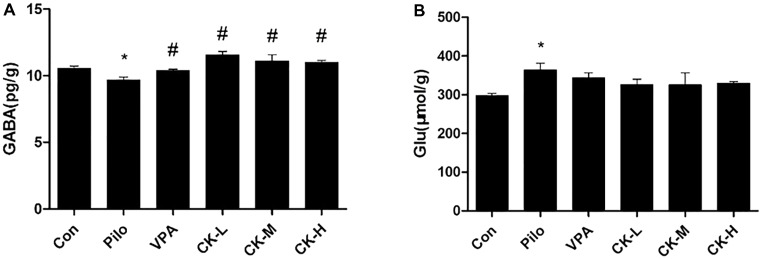
Effects of GCK on the hippocampal neurotransmitters glutamate and γ-amino butyric acid (GABA) contents in pilocarpine-induced epileptic rats. **(A)** The contents of GABA in the Con, Pilo, VPA, and GCK-treated groups. **(B)** The contents of glutamate in the Con, Pilo, VPA, and GCK-treated groups. Values are mean ± SEM (*n* = 5). Pilo 30 mg/kg, VPA 400 mg/kg, GCK 80 mg/kg, 160 mg/kg, and 320 mg/kg. Compared with the Con ^∗^*P* < 0.05; compared with the Pilo, ^#^*P* < 0.05.

To determine if GCK regulates the levels of amino acid neurotransmitter, glutamate and GABA were detected with either colorimetry or ELISA. Pilocarpine increased the content of glutamate (**Figure 3B**, *P* < 0.05), whereas GABA was declined in the hippocampus of the pilocarpine-induced epileptic rats (**Figure 3A**, *P* < 0.05). Pre-treatment with various doses of GCK (80, 160, and 320 mg/kg) and VPA (400 mg/kg) eliminated the pilocarpine-induced decreases in GABA levels (**Figure 3A**, *P* < 0.05). However, the hippocampal glutamate was not significantly affected by GCK and VPA. These observations revealed that the augmentation of GABA in the hippocampus could contribute to the anti-seizure effect of GCK.

### Effects of GCK on the Expression of GABA_A_Rα1 and NMDAR1 Protein in Pilocarpine-Induced Epileptic Rats

**FIGURE 4:**
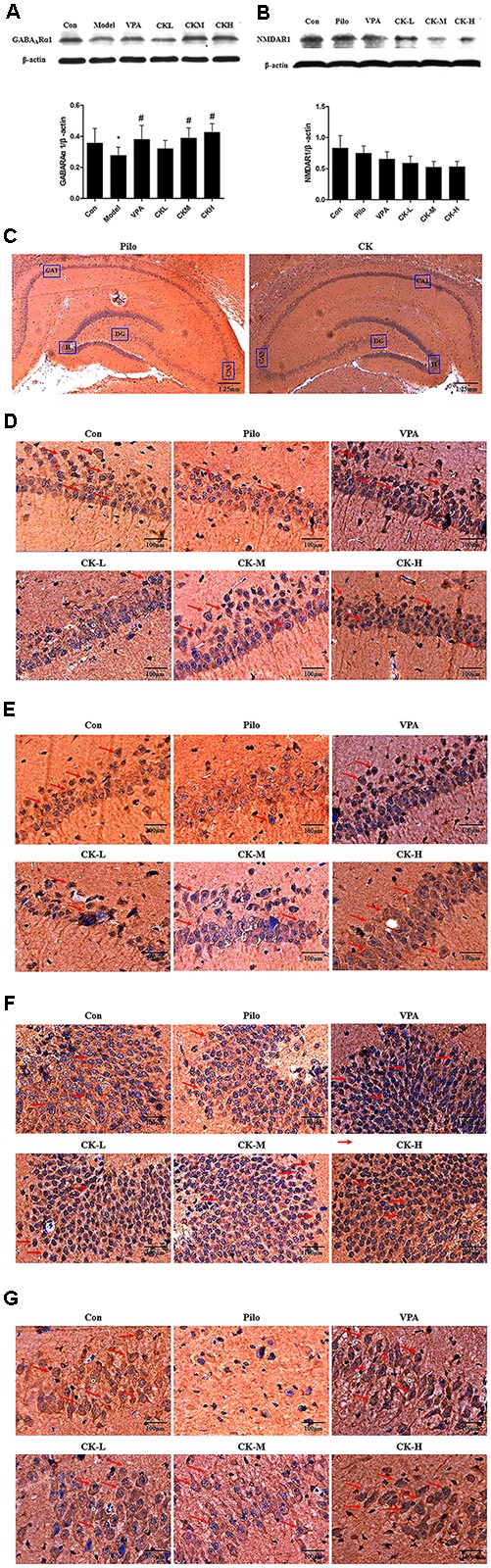
Effects of GCK on the expression of GABA_A_Rα1 and NMDAR1 protein in the hippocampus of the pilocarpine-induced epileptic rats. **(A)** The expression of GABA_A_Rα1 in the Con, Pilo, VPA, and GCK-treated groups. **(B)** The expression of NMDAR1 in the Con, Pilo, VPA, and GCK-treated groups. **(C–G)** The GABA_A_Rα1 immunoreactivity in the hippocampal CA1, CA3, DG, and H regions. Scale bar: 100 μm. Values are mean ± SEM (*n* = 5). Pilo 30 mg/kg, VPA 400 mg/kg, GCK 80 mg/kg, 160 mg/kg, and 320 mg/kg. Compared with the Con ^∗^*P* < 0.05; Compared with the Pilo, ^#^*P* < 0.05.

Glutamate acts on NMDAR to induce postsynaptic depolarization which is essential for neuronal excitability. GABA_A_R is crucially involved in GABA-mediated inhibition. The effect of GCK in the expression of receptors was also investigated. As showed in **Figure 4A**, pilocarpine injection resulted in the downregulation of GABA_A_Rα1 protein (*P* < 0.05). GCK displayed a significantly increased expression of GABA_A_Rα1 in the hippocampus (**Figure 4A**, *P* < 0.05). GABA_A_Rα1 immunohistochemical staining was distributed in all regions of the hippocampus. GABA_A_Rα1 was located in the membrane of neurons. In the pilocarpine treatment group, the staining in the sections displayed pale yellow and the positive area of GABA_A_Rα1 was smaller than those in the control group, suggesting that the GABA_A_Rα1 immunoreactivity was reduced in the hippocampus in pilocarpine-induced epileptic rats. The sections showed brown and the positive area was larger in the VPA treatment group. Furthermore, the middle and high doses of GCK could also increase the staining intensity and enlarge the positive area of GABA_A_Rα1 (**Figures 4C–G**), the results suggest that GCK also increased the GABA_A_Rα1 immunoreactivity following SE. The expression changes of NMDAR1 has a tendency to decline in a dose-dependent manner within the GCK pre-treated group although there was no significant change in NMDAR1 protein’s expression (**Figure 4B**). GCK may regulate GABA_A_Rα1’s expression to demonstrate its antiepileptic effect.

### Effects of GCK on the Expression of KCC2 and NKCC1 Protein in Pilocarpine-Induced Epileptic Rats

**FIGURE 5:**
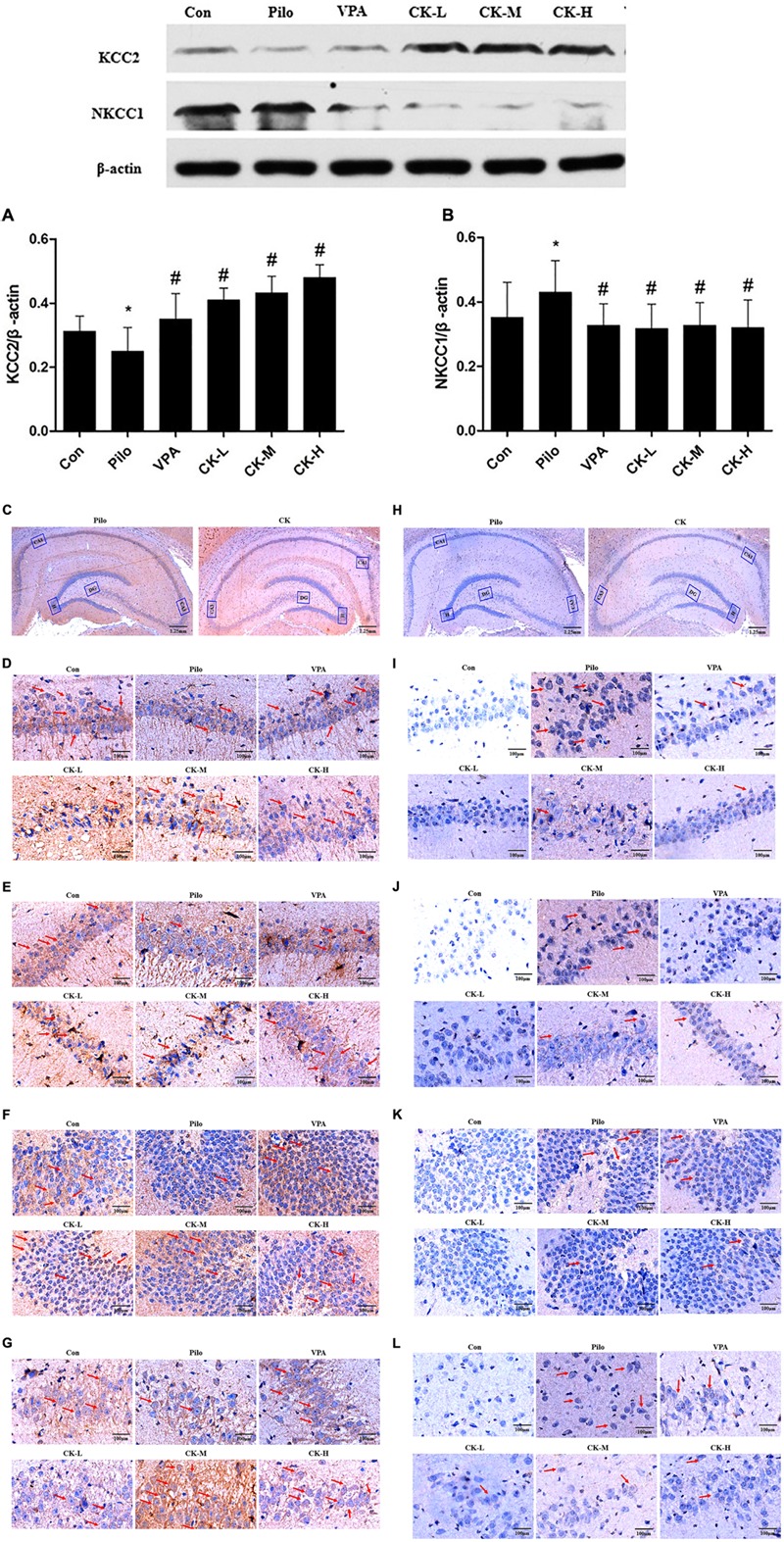
Effects of GCK on the expression of KCC2 and NKCC1 protein in the hippocampus of the pilocarpine-induced epileptic rats. **(A)** The expression of KCC2 in the Con, Pilo, VPA, and GCK-treated groups. **(B)** The expression of NKCC1 in the Con, Pilo, VPA, and GCK-treated groups. **(C–G)** The KCC2 immunoreactivity in the hippocampal CA1, CA3, DG, and H regions. Scale bar: 100 μm. **(H–L)** The NKCC1 immunoreactivity in the hippocampal CA1, CA3, DG, and H regions. Scale bar: 100 μm. Values are mean ± SEM (*n* = 5). Pilo 30 mg/kg, VPA 400 mg/kg, GCK 80 mg/kg, 160 mg/kg, and 320 mg/kg. Compared with the Con ^∗^*P* < 0.05; compared with the Pilo, ^#^*P* < 0.05.

GABA_A_R-mediated action depends on the concentration of intracellular Cl^-^. The expression of the cation chloride cotransporters, including NKCC1 and KCC2, were determined to further explain the mechanism of GCK against seizure. Western-blot analysis showed that GCK enhanced markedly the levels of the KCC2 protein in the hippocampus of pilocarpine-induced epileptic rats (**Figure 5A**, *P* < 0.05). Immunohistochemical staining showed that the KCC2 was mainly located in the membrane of neurons throughout the entire hippocampus. The KCC2 immunoreactivity was decreased in the pilocarpine-induced epileptic rats, suggesting that epileptic seizures could reduce the contents of KCC2 protein. The middle and high doses of GCK thickened the positive area of KCC2 around the surface of neurons in the hippocampus, it means that GCK increased the KCC2 immunoreactivity throughout the hippocampus (**Figures 5C–G**). GCK obviously reduced the pilocarpine-induced increased expression of NKCC1 protein in the hippocampus (**Figure 5B**, *P* < 0.05). We also observed that NKCC1 was distributed in the membrane of neurons. The positive expression of NKCC1 was very low in the hippocampus in the control group, while its staining intensity was stronger and the positive area was increased after pilocarpine induction. Interestingly, GCK significantly reduced the NKCC1 immunoreactivity in the hippocampus (**Figures 5H–L**). GCK regulates the cation chloride cotransporters to obtain an anti-seizure effect.

## Discussion

Ginsenoside compound K is a primary metabolite of ginsenoside Rb1, Rb2, and Rc in intestinal bacteria in the organisms following oral administration. Previous studies has revealed that GCK greatly improves memory impairment by inducing Nrf2-mediated antioxidant enzymes (Seo et al., 2016), attenuating cyclophosphamide-induced the deletion of hippocampal neurogenesis, and ameliorating Aβ (25–35) induced axonal atrophy and synaptic loss (Tohda et al., 2004; Hou et al., 2013). GCK could also suppress microglial activation to prevent brain impairment following cerebral ischemia (Park et al., 2012). These findings demonstrate that GCK has a beneficial neuroprotective effect in the treatment of neurological disorders (Oh and Kim, 2016). However, the anti-epileptic effect of GCK remains unknown. We found that GCK could reduce the seizure activity by increasing the content of GABA and enhancing the GABA_A_R-mediated inhibitory neurotransmission in the hippocampus.

Epileptic seizures are caused by the disruption of the balance between excitatory and inhibitory neurotransmitters (Clynen et al., 2014). GABA is a key inhibitory neurotransmitter in the brain that was decreased after seizure. The deficiency of GABA would induce neuronal hyperexcitability, which contributed to the occurrence of seizures. PTZ is a GABA_A_ receptor antagonist that acts as a chemical convulsant to build an animal model of generalized tonic-clonic seizures, myoclonic seizures, or absence seizures (Zhao et al., 2011). The rodent PTZ model is commonly used for the antiepileptic drug screening and studying the mechanism of seizures. Ethosuximide, trimethadione and valproate were discovered using this seizure model, so the PTZ test is recognized as a primary antiepileptic drug screening model by The Anticonvulsant Screening Program (ASP) of the United States National Institute of Neurological Disorders and Stroke (NINDS) (Loscher, 2017). Here we chose the PTZ-induced seizures model to test the anticonvulsant actions of GCK. We found that GCK reduced the intensity of seizures, prolonged the latency of seizures, and shortened the seizure duration in the PTZ-induced seizure rat model. Previous studies demonstrated that the Rb extract, the Mix1 (Rb1 plus Rb3) or the Mix2 (Rb1 plus Rb3 plus Rd) increases the latency to seizure onset and shortened the seizure duration in the PTZ-induced seizures rat model (Lian et al., 2005, 2006). Another study found that Rb1 dose-dependently reduces PTZ-induced seizure duration and prolongs seizure latency (Shi et al., 2018). Although these studies yielded positive results, the route of administration and the unspecified active ingredient limits its development and application. In this study, an oral administration of the gavage was performed to confirm that GCK inhibited PTZ-induced seizures. There were several causes of the beneficial effects of high doses of GCK. The intraperitoneally injection of higher doses of PTZ were used to induce a rat model of seizures, where GCK had poor water solubility and low bioavailability. These results implied that GCK could suppress myoclonic seizures and absence seizures.

Pilocarpine is an M muscarinic receptor agonist that can establish SE and partial seizures with secondary generalization (Kandratavicius et al., 2014). It is the most commonly used model of TLE, due to the appearance of spontaneous recurrent seizures, extensive brain damage, and resistance to the current antiepileptic drugs in this model (Curia et al., 2008). Lithium pre-treatment not only lowers the mortality rates, but also reinforces the epileptogenic action of pilocarpine (Martin and Pozo, 2006). In order to determinate the antiepileptic effect of GCK, we applied the lithium-pilocarpine model of epilepsy with GCK administration, where it was observed that GCK reduced the intensity of seizures and lengthened the latency to the onset of SE. These findings indicated that GCK might inhibit SE or complex partial seizures.

Alterations in the balance between Glutamate and GABA in brain could cause the occurrence and progression of seizures. Elevation of extracellular glutamate mediated neuronal excitation is generally considered as a critical factor in the pathological process of epilepsy. A growing body of evidence demonstrates a marked increase in glutamate concentration in patients with TLE (During and Spencer, 1993). Increased extracellular glutamate is strongly associated with decreased epileptogenic hippocampal volume in patients with drug resistant epilepsy (Cavus et al., 2008). The raised glutamate was found in various animal epilepsy models (Soukupova et al., 2015). In the present study, we also showed a high concentration of hippocampal glutamate. Previous studies have demonstrated that GCK attenuated glutamate-induced cytotoxicity in HT22 cells by inducing Nrf2-mediated antioxidant enzymes (Seo et al., 2016). Our experimental results showed that GCK had no significant effect on pilocarpine induced glutamate levels. Glutamate regulates brain excitability by activating the two main ionotropic receptors including NMDA and AMPA receptors. Glutamate regulates postsynaptic depolarization and action potential by binding with the NMDA receptor. Nevertheless, excess glutamate released in the synaptic cleft or the over-activated NMDA receptor promotes calcium entering, which leads to neuronal death and neurodegeneration (Deshpande et al., 2008). GCK inhibits the expression and the activity of NMDAR (Jang et al., 2004). Therefore, we explored the role of GCK in NMDAR1 expression in the epileptic rat model, which revealed that GCK exhibited a tendency to decrease the expression of NMDAR1 protein in the hippocampus. There was no obvious difference in the results, which could be caused by the small sample size. A study has showed that GCK attenuated morphine-induced dependence by decreasing the NMDAR1 expression in the frontal cortical regions of the rat brain (Yayeh et al., 2016). The cause for the inconsistency of the results could be explained by the differences between brain regions, animal models and body conditions. Another study showed that ginseng total saponins and ginsenoside Rg3 decreases the intracellular Ca^2+^ level and the hippocampal neurons death by suppressing NMDAR-induced spontaneous recurrent epileptiform discharges (Kim and Rhim, 2004). We have not assessed the function of NMDAR after GCK treatment. Thus, the effects of CK on the glutamate-mediated neuronal excitability in epilepsy requires further study.

Loss of GABA release or abnormal synthesis that impairs GABA-mediated inhibition could also facilitate neuronal hyperexcitability, which ultimately triggers seizures. Promotion of GABA release to enhance the GABA-mediated inhibitory action has become an important target of antiepileptic drugs (Kammerer et al., 2011). GABAergic interneurons and basal GABA outflow were lowered during the latent period and the initial spontaneous seizure in the pilocarpine-induced TLE (Soukupova et al., 2014). A study *in vitro* found that GCK enhances spontaneous GABA release by elevating intraterminal Ca^2+^ concentration (Bae et al., 2010). The current study demonstrated that GCK increased the level of GABA in the hippocampus. Ginsenosides could not only raise GABA levels but could also reduce glutamate levels in the hippocampus and cortex in the rat model of Alzheimer’s disease (Zhang et al., 2016). Although the models were different and the active ingredients were unclear, the results confirmed that GCK is a main metabolite of ginsenosides that could promote GABA release. These findings demonstrated that GCK could increase GABA release to suppress epileptic seizures.

γ-aminobutyric acid exerts its neuronal inhibitory via activating GABA_A_Rs-mediated inhibitory postsynaptic currents. GABA_A_Rs are identified as heteropentameric ion channels formed by 19 subunits (Uusi-Oukari and Korpi, 2010). The physiological, pharmacological, and targeting properties of GABA_A_Rs were determined by the subunit composition of GABA_A_R. The γ subunit at the synaptic sites mediates rapid phasic inhibition while the extrasynaptic δ subunit regulates the persistent tonic inhibition (Gonzalez et al., 2013). The α subunit is an important and common subunit of GABA_A_Rs. GABA_A_Rα consists of six isoforms including α1-α6. GABA_A_Rα1 is the most commonly expressed in the brain (Laverty et al., 2017). GABA_A_Rα1 is the target of benzodiazepines, which have anticonvulsant and sedative effects (Rudolph et al., 1999; McKernan et al., 2000). Multiple bodies of evidence have shown that the mutation of the α1 subunit is closely associated with several types of seizures, including early infantile epileptic encephalopathy, childhood absence epilepsy and juvenile myoclonic epilepsy (Braat and Kooy, 2015). Previous studies have shown that the expression of α1 subunit was decreased in epileptic rats (Brooks-Kayal et al., 1998). The current study also observed reduced expression of GABA_A_Rα1 in the hippocampus 1 day post-SE, suggesting GABA_A_Rα1 changed during the development of epilepsy. An earlier study showed that GCK enhanced the action of GABA receptor agonist in the brain (Jang et al., 2004). The detailed mechanism of GCK’s action remains unclear. The current study demonstrated that GCK up-regulated the expression of GABA_A_Rα1 in the hippocampus after SE. Genetic conditional enhanced expression of GABA_A_Rα1 in the DG prolonged the latency to the initial spontaneous seizure and inhibited the development of spontaneous seizures after SE (Raol et al., 2006a). GCK may inhibit the development of chronic epilepsy as a pharmacological regulator of GABA_A_Rα1 expression. Although we did not explore the effect of GCK on other GABA_A_R subunits, the elevation of GABA_A_Rα1 could offer a partial explanation of the neuroprotective effect of GCK against epilepsy.

GABA_A_R-mediated synaptic transmission was determined by the intracellular Cl^-^ concentration. Cation-chloride cotransporters plays a crucial role for maintaining Cl^-^ homeostasis (Loscher et al., 2013). NKCC1 is a main driver that promotes Cl^-^ into cells while KCC2 is a key efflux pump that mediates Cl^-^ extrusion in neurons. NKCC1 regulates the depolarizing responses to GABA_A_ receptor activation by elevating the intracellular Cl^-^ level. During the development of brain, NKCC1 expression decreases from postnatal week to maturity. KCC2 enhances fast hyperpolarizing GABA_A_ receptor-mediated inhibition by lowering the intracellular Cl^-^ concentration. Its expression increases in brain with age and maturity (Puskarjov et al., 2014a). The balance between NKCC1 and KCC2 is destroyed in the patients with TLE (Eftekhari et al., 2014). The up-regulated NKCC1 expression has been found in the brain of epileptic patients with malformations of cortical development and hippocampal sclerosis (Sen et al., 2007). Its up-regulation is also reported in the amygdala kindling model and the pilocarpine-induced epileptic animal model (Okabe et al., 2002; Li et al., 2008). Previous literature found that NKCC1 accelerates neonatal seizures in the developing hippocampus (Dzhala et al., 2005). The NKCC1 inhibitor bumetanide represents anti-seizure activity and restores the anti-epileptic effects of diazepam and phenobarbital (Dzhala et al., 2008). Bumetanide has been found to reduce the development of pharmacoresistant epilepsy (Sivakumaran and Maguire, 2016). A previous study reported that seizure frequency and epileptiform discharges are reduced in patients with TLE following bumetanide administration (Eftekhari et al., 2013). NKCC1 is a potential target for the treatment of epilepsy. In the present study, we found that pilocarpine induced the upregulation of NKCC1 protein in epileptic rats. GCK could reduce NKCC1 expression in the hippocampus in the pilocarpine-induced epileptic rats. The results showed that GCK could lower the intracellular Cl^-^ concentration to suppress GABA_A_R-mediated neuronal excitation.

KCC2 is a major impact factor of GABA-mediated hyperpolarizing postsynaptic inhibition. The expression level of KCC2 protein in the cell surface, as well as its phosphorylation state controls KCC2 function (Moore et al., 2017). Decreased KCC2 expression has been reported in human focal cortical dysplasia (Shimizu-Okabe et al., 2011). Its reduction was also observed in the animal model of pilocarpine-induced SE and brain injury-induced epilepsy (Bonislawski et al., 2007; Pathak et al., 2007). The KCC2-deficient mice exhibited spontaneous generalized seizures and were more vulnerable to seizures induced by PTZ (Tornberg et al., 2005). Two variants of KCC2, R952H, and R1049C, were discovered in human idiopathic generalized epilepsy and febrile seizures (Kahle et al., 2014; Puskarjov et al., 2014b). The loss of KCC2 impairs neuronal Cl^-^ extrusion leading to seizure-like ictal discharge (Buchin et al., 2016). CLP257 enhances KCC2 activity, which could mediate ictogenesis (Gagnon et al., 2013; Hamidi and Avoli, 2015), this suggests KCC2 is a viable therapeutic target for epilepsy. The current study found that GCK increased the pilocarpine-induced lowered KCC2 expression. These results suggested that GCK could correct the imbalance between NKCC1 and KCC2 expression to enhance GABA-mediated neuronal inhibition.

## Conclusion

Ginsenoside compound K reduced the severity of epileptic seizures and prolonged the latency to the initial seizure. GCK increased the contents of GABA in the hippocampus. Moreover, GCK up-regulated the expression of GABA_A_Rα1 and KCC2 protein while GCK reduced the pilocarpine-induced increased NKCC1 expression. Our study results suggested that GCK could inhibit the seizure activity by promoting GABA release from the hippocampal neurons and enhancing the GABAergic inhibition-related protein expression, which could demonstrate a promising future for the development of novel anti-epileptic drugs.

## Author Contributions

D-sO and KH conceived and designed the study. XZ, LC, LZ, WL, CL, WZ, SC, and QG performed the experiments. DJ and GZ provided an experimental support. XZ wrote the paper. KH helped in modifying the article. D-sO, KH, XL, and HZ reviewed and edited the manuscript. All authors gave their final approval for the submission of the manuscript.

## Conflict of Interest Statement

The authors declare that the research was conducted in the absence of any commercial or financial relationships that could be construed as a potential conflict of interest.

## Abbreviations

GABA: γ-aminobutyric acid
GABA_A_Rα1: GABA_A_ receptor subunit α1
GCK: ginsenoside compound K
KCC2: K-Cl cotransporter isoform 2
NKCC1: Na-K-2Cl cotransporter isoform 1
NMDAR: *N*-methyl-D-aspartate receptor
SE: status epilepticus
TLE: temporal lobe epilepsy.
